# Supplementary material for: A machine learning framework for computationally expensive transient models
Source: Sci Rep. 2020 Jul 13;10:11492. doi: 10.1038/s41598-020-67546-w (PMC7359323; doi:10.1038/s41598-020-67546-w)
Supplement: Supplementary file 1 — Supplementary file1 [file 41598_2020_67546_MOESM1_ESM.docx]

Supplementary Information

# A machine learning framework for computationally expensive transient models

Prashant Kumar^1^, Kushal Sinha^2^*, Nandkishor Nere^2^, Yujin Shin^3^, Raimundo Ho^2^, Ahmad Y. Sheikh^2^, Laurie Mlinar^2^

^1^Analysis Group, Boston, Massachusetts, USA

^2^Process Research and Development, AbbVie Inc., North Chicago, Illinois, USA

^3^Abbott Laboratories, Lake Bluff, Illinois, USA

^*^Corresponding Author: [kushal.sinha@abbvie.com](mailto:kushal.sinha@abbvie.com)

## **S.1 DEM Model Formulation**

Discrete Element Method (DEM) is used to study solid particle dynamics and it is a popular method to study mechanics of powders and flow of granules frequently encountered in mining, agricultural, pharmaceutical, food, chemicals and other industries. DEM describes particle motion in a Lagrangian framework wherein, equations of motion are solved for each particle or each particle acts as a computational node. At each time step, the forces acting on a particle are computed. These forces can include a multitude of forces at the granular particle scale such as friction, contact plasticity, cohesion, adhesion, liquid bridging, gravity and electrostatics depending upon the system under study. Newton’s second law of motion is solved to calculate new velocities and thus positions of the particles. Key challenges of DEM include difficulties to detect particles in contact^1^, inefficient algorithm to account for large number of particles and the inadequate modeling of interaction forces.


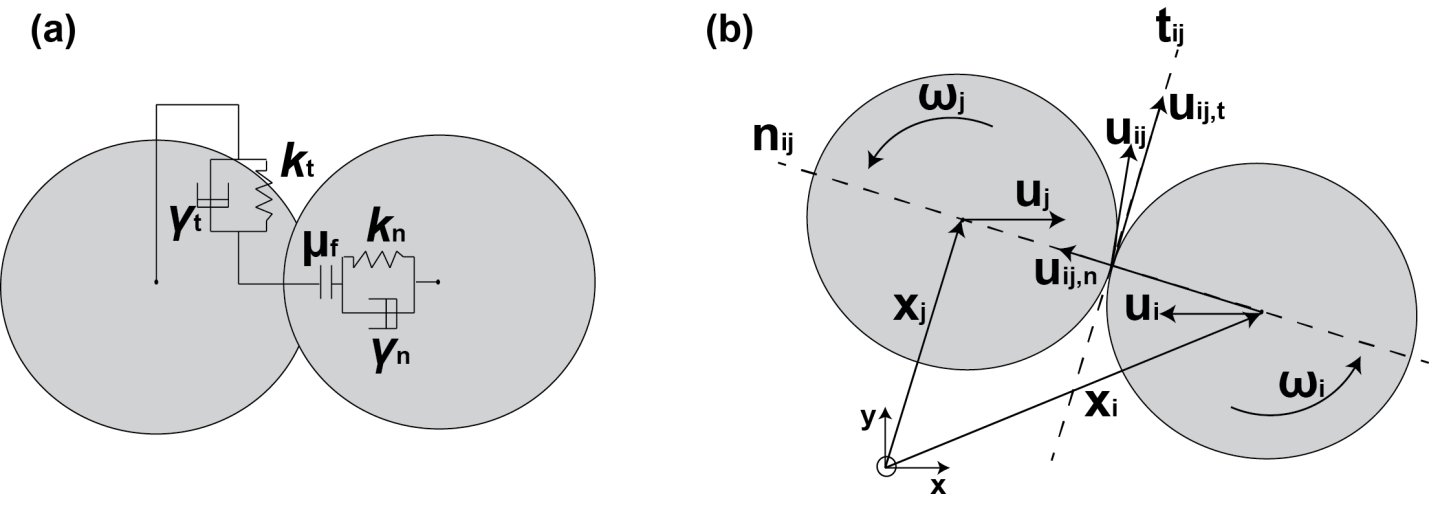


**Figure S1: a)** Schematic representation of spring-dashpot model for colliding particles, **b)** Notation and reference frame for colliding particles.

In granular systems, net force **F**_i_ exerted on a particle *i* can be decomposed into particle-particle interactions and the external forces and can be written as:

$\boldsymbol{F}_{\boldsymbol{i}}\boldsymbol{=}\boldsymbol{F}_{ex\boldsymbol{t,i}}\boldsymbol{+}\sum_{\boldsymbol{j\neq i}} \boldsymbol{F}_{ij}\boldsymbol{,}$ Eq. S.1 .1

where **F**_ij_ is the contact force exerted by *j*^th^ contacting particle on particle *i* and **F**_ext,i_ is the external force acting on particle *i.* The external force most commonly experienced by particles is the force of gravity, and was included in this work as well. There can be other effects such as hydrodynamic forces, electrostatic forces and temperature changes, which can be taken into account depending upon the problem statement. These forces are, however, neglected in this work. DEM employs soft-sphere model originally proposed by Cundall and Strack^2^. During collision, deformation is modeled as an overlap between the colliding particles, and contact force is a function of an overlap. The contact force **F**_ij_ can be further decomposed in the normal force, F_n_, and tangential force, F_t_, components that represent the compressive and shearing forces, respectively.

$\boldsymbol{F}_{ij}=F_{n}\boldsymbol{n+}F_{t}\boldsymbol{t}$*,* Eq. S.1.2

where (**n**, **t**) are the normal and tangential unit vectors. The linear spring-dashpot model was employed, as can be seen in Figure S1 (a), where the normal force is modeled as a linear spring with a damping force that opposes the relative velocity of contact for the duration of contact^2^.

$F_{n}= -k_{n}\delta_{n}-\gamma_{n}\dot{\delta_{n}}$*,* Eq. S.1.3

$F_{t}= -k_{t}\delta_{t}-\gamma_{t}\dot{\delta_{t}}$*,* Eq. S.1.4

where $k_{n}$ and $k_{t}$characterizes the stiffness of the normal and tangential spring respectively; $\delta_{n}$ and $\delta_{t}$are the normal and tangential components of overlap between particles and $\gamma_{n}$ and $\gamma_{t}$ are the viscous damping of the material in normal and tangential directions. $\dot{\delta}_{n}$ and $\dot{\delta}_{t}$are the time derivative of normal and tangential components of overlap. To avoid attractive forces, $F_{n}$ should be ensured to be non-negative. $F_{t}$ is truncated to withhold Coulomb’s friction law; |$F_{t}|\leq\mu_{f}F_{n}$, where $\mu_{f}$ is the friction coefficient. The Hertz-Mindlin model^3^ was employed to obtain the stiffness and viscous coefficients as a function of overlap and material properties such as Young’s modulus E, shear modulus G, Poisson’s ratio υ, coefficient of restitution *e*, particle radius R and particle density ρ. The Hertz-Mindlin model has no dependence on impact velocity. It has been shown to be more accurate and realistic for calculating contact forces^4^. For rolling friction we used constant directional torque (CDT) model^5^, where a constant torque is applied on the particles in the direction against relative rotation between the contacting particles. In our formulation, we treat particles as cohesive material. The simplified JKR – Jhonson-Kendell-Roberts (SJKR) model was employed, which adds an additional normal force contribution to account for the cohesive energy density of the particles, γ_cohesion_^6^. The contact area of colliding particles is approximated as a function of the overlap$\delta_{n}$^7^.

## **S.2 ARIMA**

An autoregressive (AR) model calculates the value of the response variable (y) at time (t) by regressing on the lagged values of the response variable (y) at previous time steps. An AR(p) model, where $y_{t}$ is regressed over its past p values is shown by Eq. S.2.1 (simplified version by Eq. S.2.2).

$y_{t}{= \mu+ \phi_{1}y}_{t-1}+\phi_{2}y_{t-2}{\ldots-\phi_{p}y}_{t-p}+\varepsilon_{t}$ Eq. S.2.1

Here, µ is the mean value of y, $\phi_{i}^{'}s$ are the coefficients for $y_{t-i}$ at time (t-i), and $\varepsilon_{t}$ is the error at time (t) defined by a normal distribution with zero mean and standard deviation σ ~ N(0,σ^2^).

A lag operator (J) can be introduced such that: $J^{k}y_{t}=y_{t-k}$ Eq. S.2.2

Hence, the AR (p) model can be simplified as:

$y_{t}=\mu+ \varepsilon_{t}+y_{t}{{\sum_{i=1}^{p} J}^{i}\phi}_{i}$ Eq. S.2.3

$y_{t}\Phi(J)=\mu+ \varepsilon_{t}$ Eq. S.2.4

Where, $\Phi\left( J \right)=$1-${{\sum_{i=1}^{p} J}^{i}\phi}_{i}$

A moving average (MA) model calculates the value of $y_{t}$ by regressing over the lagged values of errors. MA (q) model can be represented as shown by Eq. S.2.5.

$y_{t}{= \mu+\varepsilon_{t}- \theta_{1}\varepsilon}_{t-1}-\theta_{2}\varepsilon_{t-2}{\ldots-\theta_{p}\varepsilon}_{t-q}$ Eq. S.2.5

Implementing a similar lag operator (as with AR (p) model) on $\varepsilon_{t}$ in Eq. S.2.5; $y_{t}$ can then be written as:

$y_{t}=\mu+ \varepsilon_{t}\Theta(J)$ Eq. S.2.6

Where, $\Theta\left( J \right)=$1-${{\sum_{i=1}^{q} J}^{i}\theta}_{i}$

Combination of AR and MA models are called ARMA models and can be represented as in Eq. S.2.7.

$y_{t}\Phi(J)=\mu+ \varepsilon_{t}\Theta\left( J \right)$ Eq. S.2.7

ARIMA models are extensions of the ARMA models primarily used when the data shows non-stationary behavior, where an additional differencing term is added to adjust for the non-stationary behavior. The differencing operation is used when $y_{t}$ is non-stationary but ${(1-J)}^{d}y_{t}$ is stationary, and this operation can be implemented once or multiple times. Here, *d* represents the order of differencing. An ARIMA (*p*, *d*, *q*) model can be represented as shown in Eq. S.2.8.

${\Phi(J){(1-J)}^{d}y}_{t}=\mu+ \varepsilon_{t}\Theta\left( J \right)$ Eq. S.2.8

A sensitivity analysis should be performed in order to identify appropriate values for ‘p’, ‘d’, and ‘q’ variables. Multiple ARIMA models were generated by sampling the variables p, d, and q uniformly between 0 and 100, 0 to 2, and 0 to 2, respectively. Given that errors at previous time steps are unobserved variables, maximum likelihood estimation (MLE) should be performed in order to find the best model. Akaike Information Criterion^8^ (AIC) score was used to select the best ARIMA model after comparing each model against other models. For a model with *n* number of fitted parameters and *L* being the maximum value of the likelihood function (defined by Eq. S.2.10), the AIC score can be defined as:

$AIC = 2n-2log(L)$ Eq.S.2.9

$\log\left( L \right)=-\frac{T-p}{2}\log\left( 2\pi\right)-\frac{T-p}{2}\log\left( \sigma^{2} \right)-\sum_{t=p+1}^{T} \frac{\varepsilon_{t}^{2}}{2\sigma^{2}}$ Eq. S.2.10

Here, T is the total number of observations.

The model with the minimum AIC score is chosen as the best model.

## **S.3 Identification of Mixing Regimes**

Apart from having an accurate knowledge of the mixing time, getting qualitative information of the rate of particle mixing can be crucial in understanding, designing and operating the drying process. Based on the initial behavior of the segregation index, two mixing regimes were identified, namely fast (less than 1 minute) and slow (longer than 1 min) mixing regimes. Cases requiring agitation time of less than or equal to 1 minute to achieve homogeneity were labeled as ‘fast’ and others as ‘slow’. Support Vector Machine (SVM) method was used to build a robust classifier in order to identify the mixing regimes for different combinations of material and equipment properties. Performance of the SVM classifier was evaluated by calculating the area under the precision recall curve (AUPR) and the area under the receiver operating characteristic curve (AUROC). SVM classifier performed well on the simulated DEM dataset for the agitated filter dryer with an AUPR of 0.95 and AUROC of 0.92 as can be seen in Figure S1.

$Precision=\frac{TP}{TP+FP}$ ; $Recall=\frac{TP}{TP+FN}$; $False Positive Rate=\frac{FP}{FP+TN}$ Eq.S.3.1

where TP: True Positive; FP: False Positive; TN: True Negative; FN: False Negative


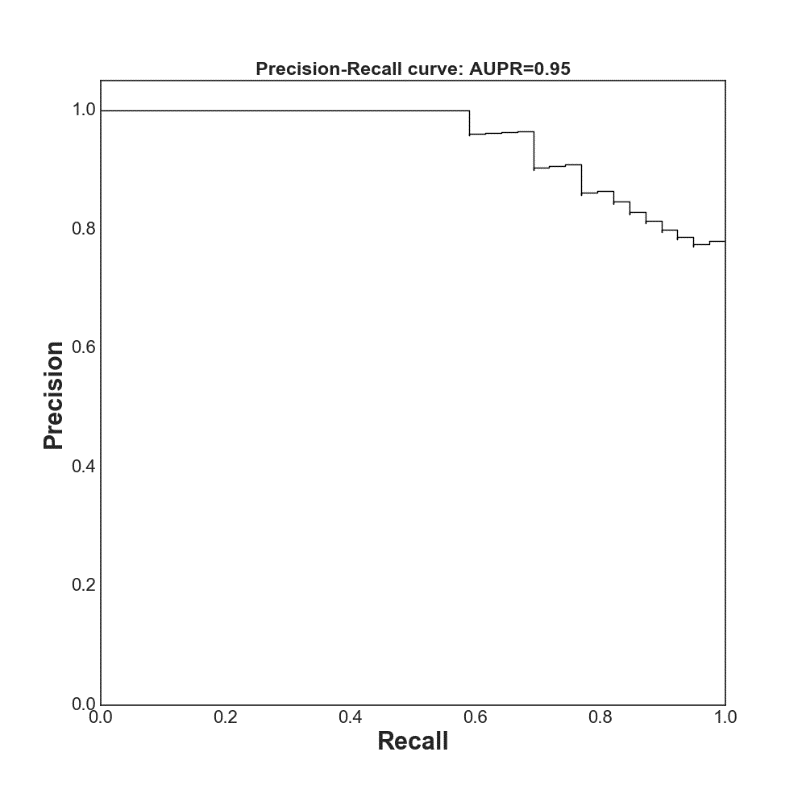

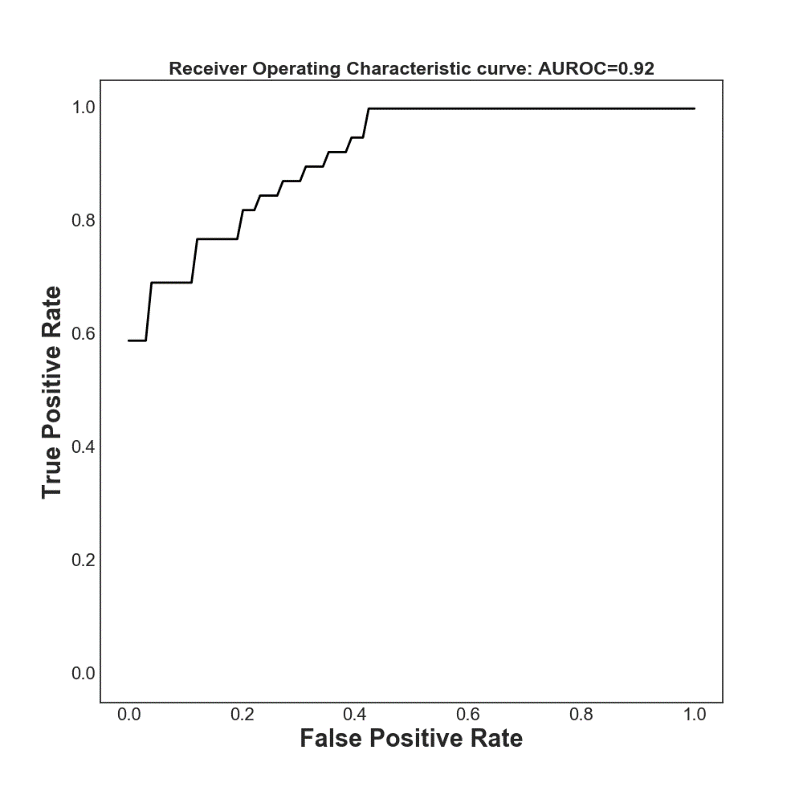


**Supplementary Figure 1: Precision-Recall (AUPR=0.95) and the Receiver Operating Characteristic curves (AUROC=0.92)**. High precision and recall shows the power of the machine learning classifiers, and this approach can assist in quick qualitative estimation of mixing time.

## **S.4 ARIMA Validation**


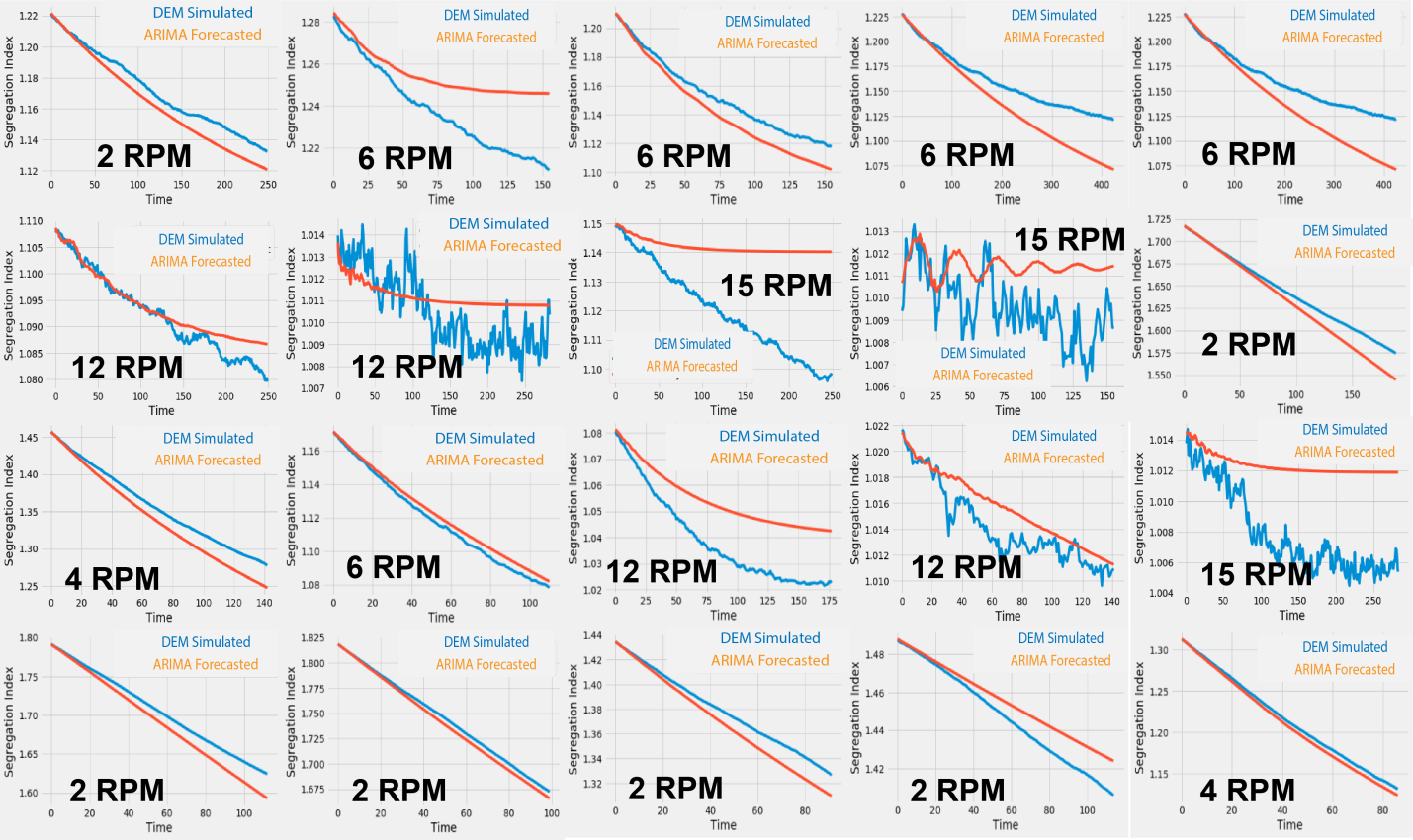


**Supplementary Figure *2*: Validation of ARIMA with DEM Simulated data for the time evolution of segregation index.** ARIMA was trained to learn the time evolution of segregation index on the first half time-steps of the DEM simulated data, and then the learned model was used to forecast and validate on the latter half of the time-steps.


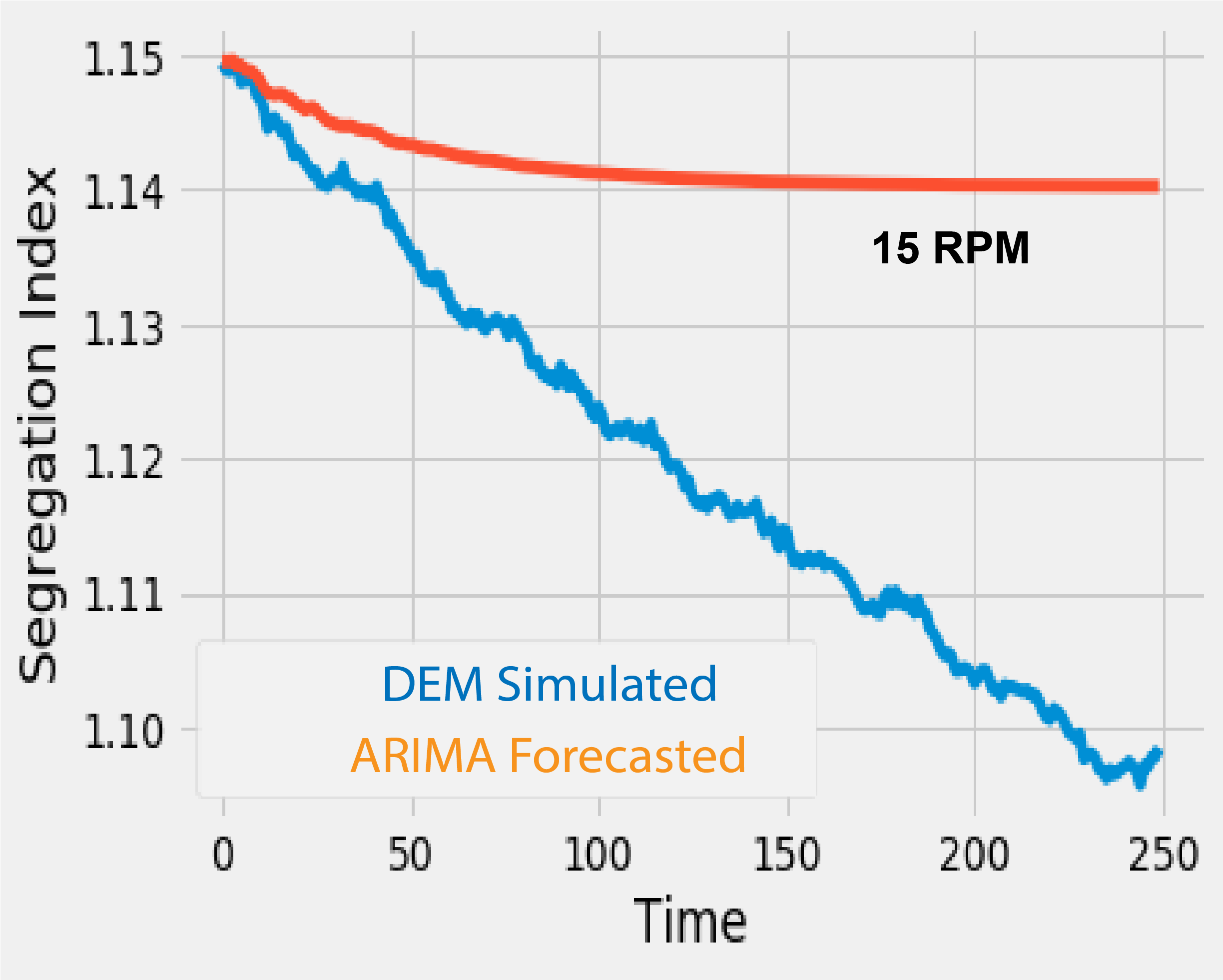


**Supplementary Figure 3: Error between ARIMA forecasting and DEM simulations is less than 5% in all the simulations.** In Supplementary Figure 2, the difference between ARIMA forecasting and DEM simulations may appear large but in reality, the error is always less than 5% which shows that the ARIMA forecasting is very robust.


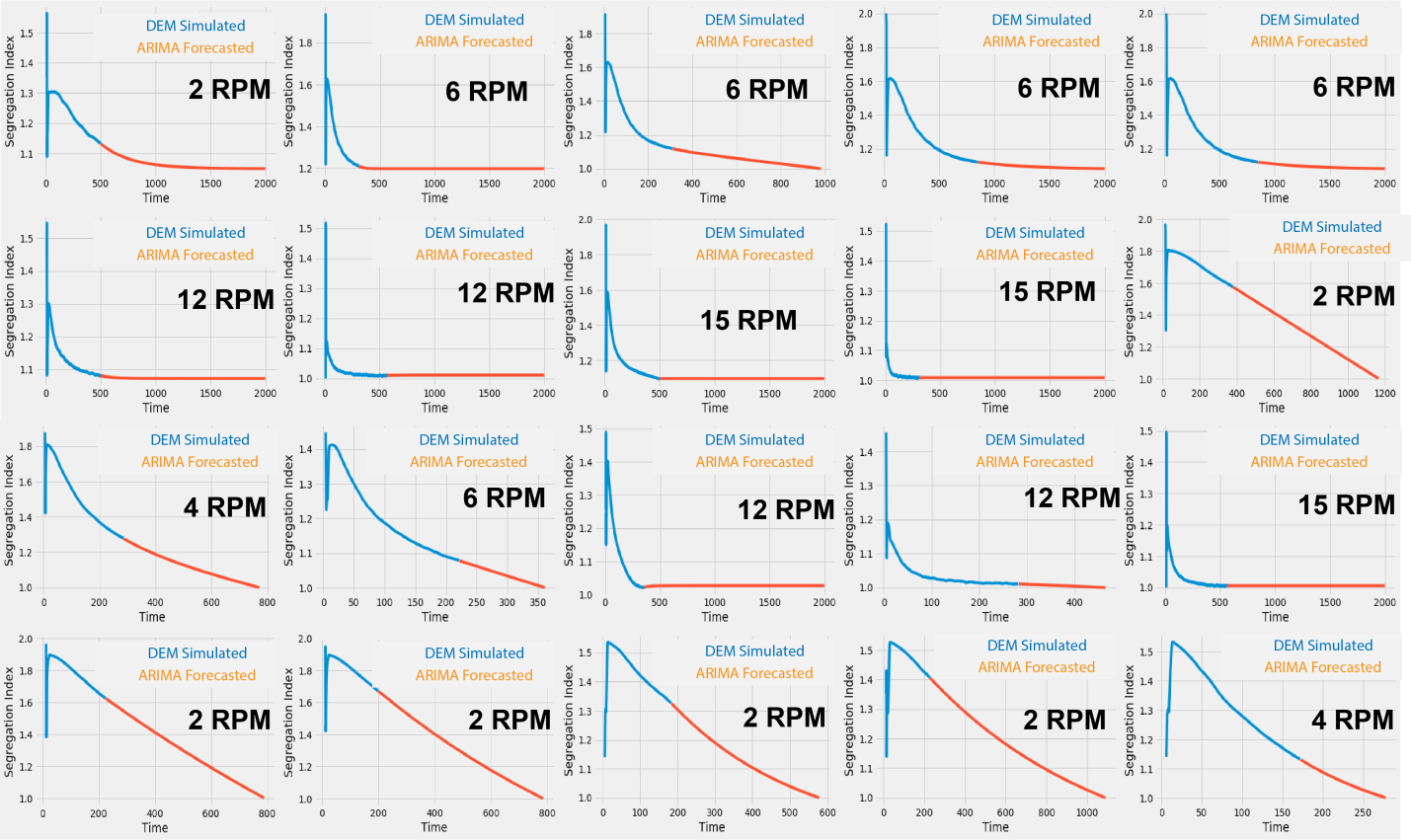


**Supplementary Figure 4: ARIMA Forecasting of the segregation index for systems with different material properties and RPM.**


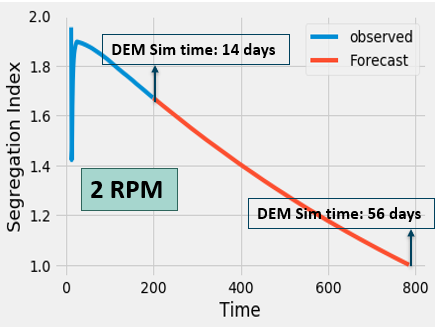


**Supplementary Figure 5: Temporal evolution of segregation index using the integrated DEM-ARIMA approach shows that for segregation index to reach 1, DEM alone would have taken approximately 56 days and this simulation time was reduced to 14 days by forecasting the segregation index using ARIMA.**

## **S.5 Performance of the machine learning methods**

**Supplementary Table 1: RMSE for different machine learning methods.** It was seen that random forest has the lowest RMSE of 16.61 seconds. Random forest performs slightly better as compared to the other tested methods as can also be seen from the coefficient of determination of the prediction.

| **Method** | **RMSE (seconds)** |
| --- | --- |
| Random Forest | 16.61 |
| Support Vector Regression | 17.78 |
| Elastic Net Regression | 17.71 |
| Partial Least Squares Regression | 18.34 |

## **S.5 Input data from simulations**

Please find the input data for ML models at following location: <https://zenodo.org/record/3746953#.Xo-l-HtOmUl>

The ML and ARIMA codes used in the paper with sample input data are located here: <https://github.com/kushalsinha/AbbVie_FilterDryer_ARIMA_ML>

# **References**

1. Adam, S., Suzzi, D., Radeke, C. & Khinast, J. G. An integrated Quality by Design (QbD) approach towards design space definition of a blending unit operation by Discrete Element Method (DEM) simulation. *Eur. J. Pharm. Sci.* **42,** 106–115 (2011).

2. Cundall, P. A. & Strack, O. D. L. A discrete numerical model for granular assemblies. *Géotechnique* **29,** 47–65 (1979).

3. Mindlin, R. D. Compliance of Elastic Bodies in Contact. *J. Appl. Math.* **71,** (1949).

4. Zhang, H. P. & Makse, H. A. Jamming transition in emulsions and granular materials. *Phys. Rev. E* **72,** 011301 (2005).

5. Ai, J., Chen, J.-F., Rotter, J. M. & Ooi, J. Y. Assessment of rolling resistance models in discrete element simulations. *Powder Technol.* **206,** 269–282 (2011).

6. Johnson, K. L., Kendall, K. & Roberts, A. D. Surface Energy and the Contact of Elastic Solids. *Proc. R. Soc. A Math. Phys. Eng. Sci.* **324,** 301–313 (1971).

7. Kloss, C., Goniva, C., Hager, A., Amberger, S. & Pirker, S. Models, algorithms and validation for opensource DEM and CFD-DEM. *Prog. Comput. Fluid Dyn. An Int. J.* **12,** 140 (2012).

8. Bozdogan, H. Model selection and Akaike’s Information Criterion (AIC): The general theory and its analytical extensions. *Psychometrika* **52,** 345–370 (1987).
